# Recent Advances in Nanostructured Conducting Polymers: from Synthesis to Practical Applications

**DOI:** 10.3390/polym8040118

**Published:** 2016-03-31

**Authors:** Duong Nguyen Nguyen, Hyeonseok Yoon

**Affiliations:** 1 Department of Polymer Engineering, Graduate School, Chonnam National University, 77 Yongbong-ro, Buk-gu, Gwangju 61186, Korea; nguyendun@gmail.com; 2 School of Polymer Science and Engineering, Chonnam National University, 77 Yongbong-ro, Buk-gu, Gwangju 61186, Korea

**Keywords:** conducting polymers, nanomaterials, nanohybrids, synthesis methods, applications

## Abstract

Conducting polymers (CPs) have been widely studied to realize advanced technologies in various areas such as chemical and biosensors, catalysts, photovoltaic cells, batteries, supercapacitors, and others. In particular, hybridization of CPs with inorganic species has allowed the production of promising functional materials with improved performance in various applications. Consequently, many important studies on CPs have been carried out over the last decade, and numerous researchers remain attracted to CPs from a technological perspective. In this review, we provide a theoretical classification of fabrication techniques and a brief summary of the most recent developments in synthesis methods. We evaluate the efficacy and benefits of these methods for the preparation of pure CP nanomaterials and nanohybrids, presenting the newest trends from around the world with 205 references, most of which are from the last three years. Furthermore, we also evaluate the effects of various factors on the structures and properties of CP nanomaterials, citing a large variety of publications.

## 1. Introduction

Over the past decade, there has been significant progress in fabricating nanostructured materials with unique properties. In particular, the topic of intrinsically conducting polymers (CPs) has seen an explosion in the number of published papers, which have been very useful for both fundamental research and potential applications. CPs are polymeric materials that display high conductivities, good electrochemical activity, unique optical properties, and biocompatibility. Because of these interesting properties, CPs have received special attention as promising candidates in many areas of nanoscience and nanotechnology.

**Figure 1:**
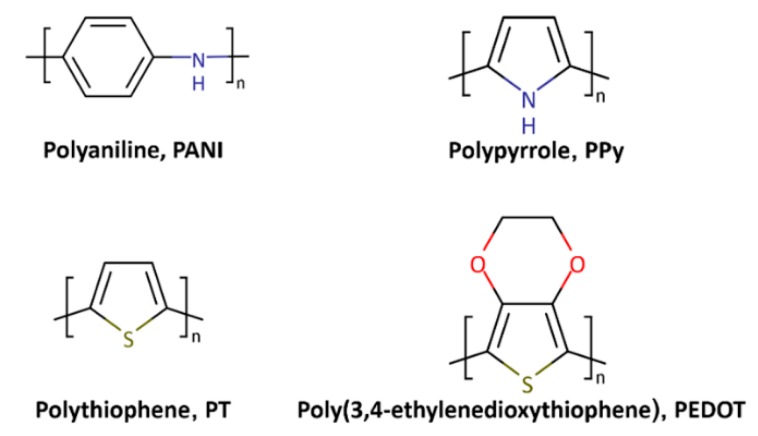
Chemical structures of representative conducting polymers (CPs).

**Figure 2:**
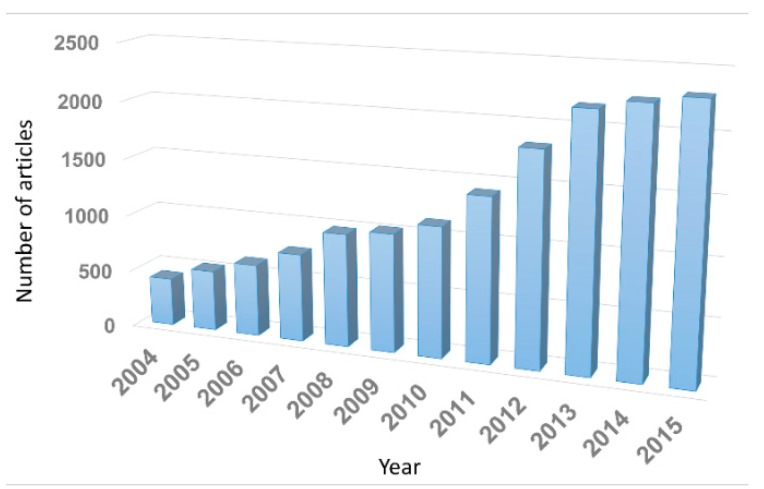
The number of articles published on CP nanomaterials over the last decade. Data from the ISI Web of Knowledge database.

CPs, also known as conjugated polymers or “synthetic metals,” are polymers with highly π-conjugated polymeric chains. Alan MacDiarmid, Hideki Shirakawa, and Alan Heeger were awarded the Nobel Prize in Chemistry in 2000 for the discovery of inherent CPs. Up to now, a variety of CPs (e.g., polyaniline (PANI), polypyrrole (PPy), polythiophene (PT), poly(3,4-ethylenedioxythiophene) (PEDOT), and other PT derivatives) have been developed (see Figure 1). While there has been continuous progress in the development of CPs, there has also been increasing interest in CP nanohybrids. Hybrid nanomaterials, the combination of CPs with different types of materials, such as metals, carbonaceous materials, and inorganic compounds, have been studied most intensively. Such nanohybrids have proven to be attractive for a wide variety of applications, from organic electronics to energy storage, solar cells, and sensors. As can be seen in Figure 2, the number of studies regarding CP nanomaterials has rapidly increased over the last decade.

From the very early years of studying CPs, there have been numerous efforts to hybridize CPs with inorganic species. Metals (Ag, Au, and Pd), metal oxides (NiO, Cu_2_O, CuO, TiO_2_, WO_3_, ZnO, Fe_2_O_3_, Fe_3_O_4_, MnO_2_, SnO_2_, V_2_O_5_, and RuO_2_), and chalcogenides (Bi_2_S_3_, CdSe, CdS, and CdTe) have been explored. Recently, carbon nanomaterials (graphene, graphene oxide, and nanotubes) have been frequently employed. This significant investment in development is due to the recognition that nanostructured CPs can play a key role in applications such as Li-ion batteries and supercapacitors (fast electrolyte diffusion), solar cells (effective exciton dissociation), sensors (enhanced sensitivity), and electrochromics (fast response times). In this review article, we first classify traditional synthesis routes for CPs, as well as for hybrid nanomaterials, and present a new standard for classification. From this point of view, we then discuss recent developments in synthesis approaches that have improved conventional methods. Finally, various selected applications have been reported in order to demonstrate the merits of CPs. The key difference between our work and previously published studies [1,2,3,4,5,6,7,8] is that we provide a brief scheme of the synthesis processes for CP nanomaterials, including the mechanism of polymerization, key rules of synthesis, and environments of reactions (e.g., solid, liquid, gas) instead of focusing on the details of one-dimensional (1D) nanostructures [2,3], electrochemical synthesis [5], or sensor applications [1,4,6,8]. This approach creates a unified view of the advantages and disadvantages of each method, thereby enabling the significant features to be better understood and, hopefully, to anticipate new synthesis routes for CP nanohybrids.

As will be shown later, different synthesis approaches can be conveniently categorized based on three particular aspects: (i) the oxidizing agent or synthetic mechanism, (ii) the template used in the synthesis reaction; and (iii) the order of addition of each component. In this review, we discuss various procedures for fabricating CP-only nanomaterials, as well as CP-based nanohybrids (binary and ternary systems), which are the root of hybrid nanomaterials.

## 2. Overview and Classification of Synthesis Methods

### 2.1. Polymerization Mechanism

**Figure 3:**
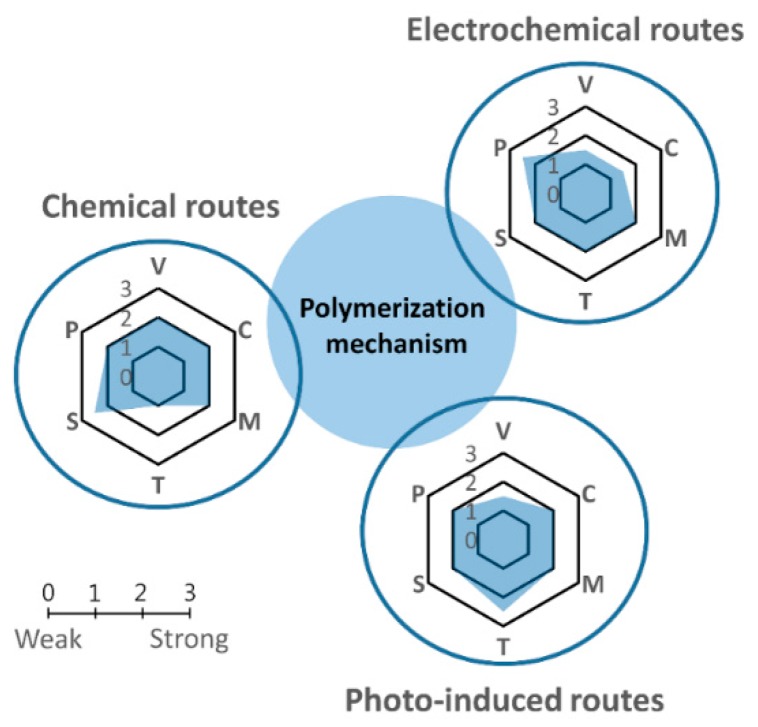
Schematic comparing the chemical, electrochemical, and photopolymerization mechanisms. Each method has been evaluated in term of variables (V), in which a low value means there are many key variables in the synthesis process; cost effectiveness (C), in which a low value corresponds to high cost; morphology control (M); time of reaction (T); scalability (S); and the resulting purity (P) of the materials.

CPs are usually synthesized via oxidative coupling of monomers. For polymerization, the first step is the oxidation of the monomer, which results in the formation of a radical cation, which then reacts with another monomer or radical cation, forming a dimer. Hence, an obvious classification is the initiation process of polymerization. The three general initiation routes are chemical, electrochemical, and photo-induced oxidation, each having its own advantages and disadvantages (Figure 3). In the first route, chemical oxidants (such as ferric chloride or ammonium persulfate) are applied to oxidize the monomer. In the second route, the monomers are oxidized electrochemically, and in the third route, light is required to oxidize the monomer with a photoinitiator. A partial summary of these techniques is shown in Table 1.

Chemical synthesis methods involve either condensation polymerization (step-growth mechanism) or addition polymerization (chain-growth mechanism). The main advantage of chemical synthesis is that it not only provides various possible routes to synthesize different CPs, it also permits the large-scale production of these materials, which is currently impossible with electrochemical synthesis. Furthermore, chemical synthesis methods have more options in terms of covalent modification of the CP backbone.

Electrochemical synthesis is a relatively straightforward synthetic method for fabricating CPs. The conductivity of CPs allows them to be electrochemically polymerized. In general, electrochemical polymerization is employed using a three-electrode system (working, counter, and reference electrodes) in a solution comprising a monomer, an electrolyte, and appropriate additives. A number of crucial factors must be considered, including the electrolyte, deposition time/method (continuous *versus* pulsed) and applied potential. Each of these parameters has an effect on film morphology, conductivity, and mechanical properties, which directly impact the utility of the material for many applications.

The significant differences between chemical and electrochemical methods have been investigated by many researchers. One difference is that very thin CP films (approximately 20 nm in thickness) can be produced using electrochemical polymerization, whereas powders or very thick films are typically produced using the chemical technique [25]. However, this idea is being challenged. Much effort has been expended on experimental research to overcome this difference, and at the moment, the chemical route can generate thin CP films via modification of the type and concentration of the oxidizing agent. Although chemical oxidative polymerization can also be applied, the electrochemical route is still preferable for thin CP films because employing an appropriate electrical potential allows the production of high-quality films with the desired thickness [26]. Some drawbacks of the electrochemical method are the relatively poor reproducibility of bulk CPs and the fact that it is quite difficult to remove the grown film from the electrode surface. Most CPs can be synthesized by chemical polymerization, but electrochemical synthesis is limited to those designs in which the monomer can be oxidized via a potential to form reactive radical ion intermediates for polymerization; several standard CPs (*i.e.*, PPy, PT, PANI, PEDOT) can be fabricated both chemically and electrochemically. To date, owing to the chemical diversity of the studied monomers, a general scheme for the electrochemical route cannot be provided. Several reports have made direct comparison of chemical and electrochemical polymerization methods for the same monomers [27,28], which interestingly show that the electrical conductivities of CPs produced electrochemically were higher than those of CPs produced via chemical polymerization. Another work by Gorey *et al.* showed that the polymerization time for the electrochemical approach is more rapid than that using chemical methods (a few minutes *versus* a few hours), whereas chemical growth can provide more homogeneous morphologies than the electrochemical route [29].

Photopolymerization (or photoinitiation) is another approach by which monomers can be polymerized by exposure to ultraviolet (UV) light, visible light, laser-generating radicals (photochemical reaction), or holes (photoelectrochemical reaction). Common examples of photopolymerization can be divided into two main categories: (i) direct photopolymerization and (ii) photosensitizer-mediated polymerization. Direct photopolymerization proceeds by absorption of the energy of illumination and then decomposition of the monomers into radicals, which is similar to free radical polymerization. However, it should be noted that CPs cannot be achieved by direct photopolymerization because they have a more positive oxidation peak potential than the redox potential of the photosensitizers [21]. On the other hand, in the photosensitizer-mediated polymerization, the energy transfer from the light occurs via the photosensitizer in order to form the corresponding excited states. In photochemical polymerization, photosensitizers can be used as photocatalysts (e.g., ruthenium complexes, silver nitrate, camphorquinone, and ketones). As compared to the conventional chemical route, photochemical polymerization is more advantageous because the radical is formed through hydrogen abstraction by irradiation, which is generally more efficient than direct fragmentation via a thermal reaction. From a thermodynamic viewpoint, this approach can resolve the problem of large activation barriers for the reaction, which is the limitation of chemical polymerization. As a consequence, the initiation rate can be very fast and well controlled by simply turning the illumination source on or off. In addition, this process provides better control over the shape, size, and physical properties of CP nanomaterials by tuning the source of the initiator, light intensity, and temperature. For photoelectrochemical polymerization, the photosensitizer is a dye-sensitized semiconductor (e.g., metal oxides such as TiO_2_, ZnO, and WO_3_; chalcogenides such as CdS, CdSe, and GaAs) or simply a dye. This approach was developed in order to solve the problems of electrochemical polymerization. One can see that in some cases, such as in infiltration of CPs into oxides (e.g., TiO_2_, SnO, W_2_O_5_, and ZnO), the electrochemical route (*i.e.*, electrodeposition) is often hindered by the low conductivity of the inorganic matrix. Consequently, high potentials must be applied, causing irreversible oxidation (an over-oxidative state) of the electrogenerated CP. Photoelectrochemical polymerization can overcome this issue because illumination of the semiconductor electrode employs band-gap irradiation and improves the electrical conductivity via photoconductivity. The mechanism of photoelectrochemical polymerization can be described as follows: when the semiconductor photosensitizer is illuminated, electrons from the valence band are stimulated to the conduction band. These electrons are drained into an external circuit, leaving holes in the solution. When the photogenerated holes react with a particular monomer, oxidation begins and initiates polymerization. A primary aspect of photoelectrochemical polymerization is that the oxidation step is guided by the difference between the potential of the valence band edge of the inorganic species (e.g., metal oxide) and the oxidation potential of the monomer. Comparing photoelectrochemically produced CPs with those prepared either chemically or electrochemically, the former is independent of the nature of the electrolyte in terms of both the generation and the electrical properties of the resulting CPs. Furthermore, photoelectrochemical polymerization allows for the deposition of CPs into micro-templates [30]. However, not all CPs can be produced by photoelectrochemical polymerization.

Overall, although chemical oxidative polymerization can produce CPs with controlled sizes and shapes, the electrochemical route provides for large-area synthesis, relatively short processing times, and good electrical, mechanical, and optical properties. Photopolymerization remains a valuable method that needs further exploration.

### 2.2. Methods for Fabricating Conducting Polymer Nanomaterials

**Figure 4:**
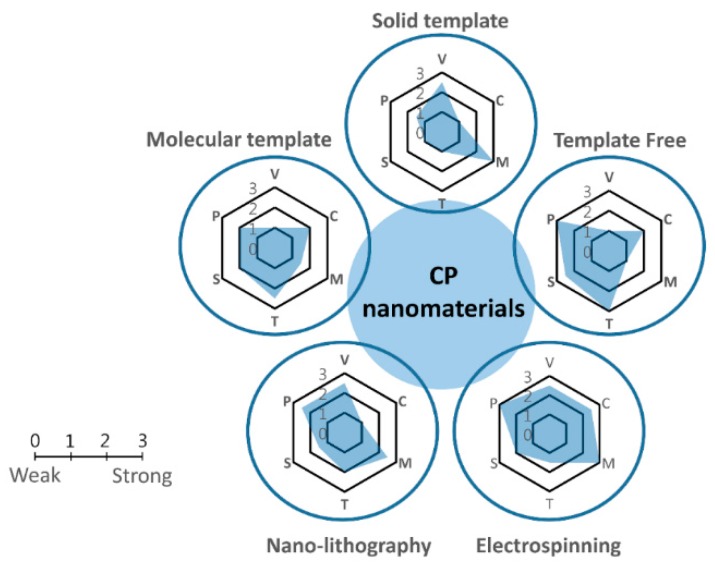
Schematic of the synthesis methods for CP nanomaterials. Each mechanism has been evaluated in term of variables (V), in which a low value means there are many key variables in the synthesis process; cost aspect (C), in which a low value corresponds to high cost; morphology control (M); time consumption (T); scalability (S); and purity (P) of the products.

Prior to discussing recent progress in the development of CP-based nanomaterials in detail, it is worth briefly categorizing and summarizing the template-based synthesis procedures employed to obtain various nanostructures. Generally, CPs with different nanostructures can be prepared by a number of methods, including microemulsion polymerization, template filling, and lithographic techniques (Figure 4). To the best of our knowledge, however, there are no distinct definitions of the methods, e.g., the difference between hard and soft templates is sometimes ambiguous. It should be noted that traditional classification is still not unified. For instance, some researchers consider the terms “template-free” and “self-assembly” as other names for “soft template” [1,2], whereas other studies arrange them into separate groups [3]. Therefore, we suggest a new standard for the classification, namely, the solid template approach, the molecular template approach, and the template-free approach (Table 2), which we defined in a previous study [4]. This classification is based on two aspects: the preparation step and the ability of the technique to achieve shape modification and stability. Each approach has its own merits and demerits. First, the solid template needs to be prepared before the synthesis of CPs (as-processed), whereas this is not necessary with molecular templating. Second, in solid templates, the shape of the CPs is mainly determined by the template, whereas for molecular templates, the morphology of the CPs can be varied by changing key synthetic conditions. Finally, solid template techniques are more stable than molecular templating for various conditions of temperature, pH, additives, *etc.* These various approaches are discussed in the following subsections.

#### 2.2.1. Solid Template Approach

The solid template method is a feasible, powerful, and controllable tool for preparing nanostructures of inorganic semiconductors, metals, and polymers. In this method, a template membrane is usually required to grow nanostructures inside pores or channels of the membrane, providing for complete control of the size and shape of the nanostructures. The solid template method is attractive as it can be used either chemically or electrochemically. For chemical template synthesis, the membrane can simply be immersed in a solution of the desired monomer, oxidant, and dopant; monomer polymerization then occurs within the pores of the membrane, which act as a template. Electrochemical template synthesis can be also accomplished by employing a metal film as an electrode at the bottom of the membrane; polymerization is subsequently carried out within the pores at an appropriate applied potential. However, because of limitations in the size and pore density of the solid template, large-scale production by the electrochemical template method is impossible. Although chemical polymerization can be controlled by adjusting the polymerization time (e.g., short time for thin-walled tubes and longer times for thick-walled tubes or fibers) and the kind and concentration of oxidizing agent, electrochemical template synthesis is more controllable via changing the applied potential or current density, as well as the deposition time and interval. The main disadvantage of the solid template method is that the template needs to be removed by post-processing, which often destroys or disorders the formed nanostructures or degrades the major properties of the CP. Polycarbonate membranes prepared by a ‘‘track-etching” method and porous alumina membranes produced by an electrochemical approach have been widely used as commercial templates.

#### 2.2.2. Molecular Template Approach

Although the solid template method gives precise control over the shape and size of CPs, it has significant drawbacks such as (1) small quantity production, (2) expensive templates; and (3) the need to use of harsh chemicals (e.g., strong acids and bases) in order to remove the template. As a result, researchers have developed molecular templates as an alternative to the solid template method. The main advantage of this approach is that it is relatively simple, inexpensive, and versatile for fabricating nanostructures. Importantly, the molecular template approach has strong potential for producing large quantities of CP nanomaterials. Many molecular templates have employed surfactants, surface micelles, liquid crystalline phases, and structure-directing molecules, which are commonly made via self-assembly mechanisms using hydrogen bonding, van der Waals forces, π–π stacking, electrostatic interactions, *etc.* The size and morphology of the final products are predominantly determined by the pre-assembled molecular templates. Therefore, it is crucial to maintain the microstructure of the molecular template during polymerization in order to obtain the desired product. Among the various molecular template routes, the surfactant-assisted approach is widely applied because surfactant meso-phases are versatile molecular templates that are arranged in regular structures through self-assembly. Cationic cetyltrimethylammonium bromide (CTAB) [31,32,33], anionic sodium 4-[4-(dimethylamino) phenyldiazenyl]benzenesulfonate (MO) [34,35], sodium dodecylsulfonic acid (SDS) [36,37,38], sodium dodecylbenzenesulfonate (SDBS) [23] and non-ionic isooctylphenyl ether (Triton™ X-100) [39,40] are conventional surfactants that have been widely used to prepare CP nanomaterials with different morphologies in several polymerization systems.

In general, the disadvantage of such a method is the poor control of the size, morphology, and orientation of the CP nanostructures [6], which has prevented wide utilization of molecular template synthesis. In order to resolve the limitations of this method (the equilibrium shape and size of surfactant aggregates), numerous factors must be considered, such as the geometry of the template molecules (for example, in the case of micelles, the length of the surfactant tail in the hydrophobic core and the volume and effective area of each surfactant head group) [1].

#### 2.2.3. Template-Free Approach

In order to circumvent the drawbacks of the template approach, the development of synthesis methods without using templates has been studied. It should be noted that the use of a template increases the cost of the synthesis and may also either modify or damage the properties of the synthesized nanostructures. Hence, a template-free method is highly attractive because a template is not required for either the synthesis or the stabilization of the nanostructures.

One of the frequently cited methods is *in situ* polymerization using protonic acids (e.g., β-naphthalenesulfonic acid) as dopants [41]. The main feature of this method is that the micelle-like structures formed by the monomer and dopant act as “molecular templates” during polymerization. The template-free approach has great potential for preparing unique micro- or nanostructures that cannot be fabricated by the solid template approach due to the limitations imposed by the pore structure of the template used. Indeed, the template-free route is attractive owing to its low cost, simplicity, and flexibility for thin-film device fabrication. Nevertheless, the mechanisms of self-assembly are quite complicated and not clearly understood. The template-free method limits precise control of the morphology and properties, a drawback that is similar to that of the molecular template approach [3].

#### 2.2.4. Other Methods

Some publications have reported other methods for producing CP nanostructures. For example, soft lithography is a low-cost, high-resolution, and high-throughput approach to fabricating nanoscale patterns using a micro-mold, sometimes with the assistance of solvent vapors or temperature control [42]. In addition, the directed electrochemical nanowire assembly technique has been used to grow CP nanowires [43]. Among the physical methods used to prepare CP nanomaterials without templates, electrospinning is one of the most popular methods because it is easy and effective for generating long CP nanofibers using strong electrostatic forces. As compared to other synthesis routes, the electrospinning process seems to be the only method that can produce continuous long nanofibers. However, to achieve good fiber formation, some non-conducting polymers (e.g., polyethylene oxide (PEO)) are usually added for the purpose of tailoring the viscosity of the mixture [44]. Furthermore, the nanofibers may exhibit a substantial concentration of defects and a large size distribution, which limit the application of electrospinning.

### 2.3. Synthesis Methods for CP Nanohybrids

**Figure 5:**
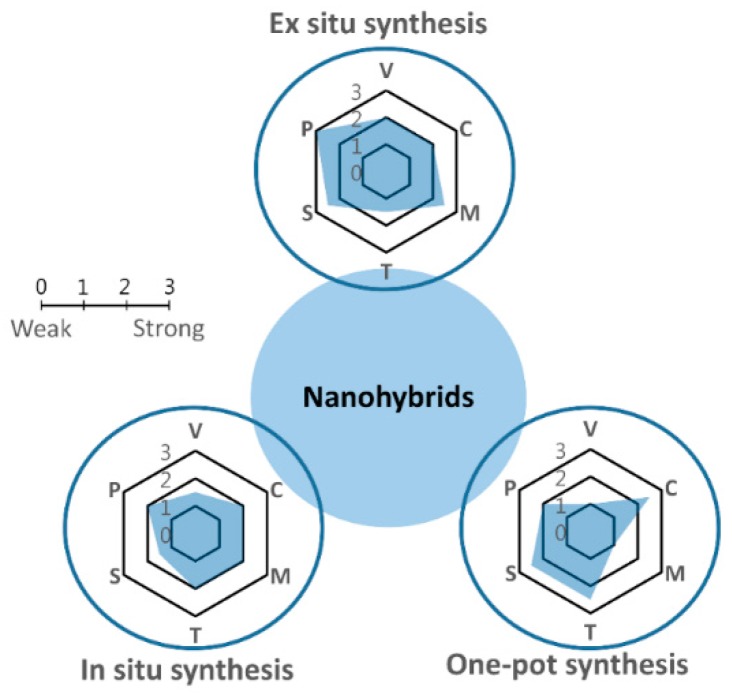
Schematic of nanohybrid synthesis methods. Each mechanism has been evaluated in terms of variables (V), in which a low value means there are many key variables in the synthesis process; cost (C), in which a low value corresponds to high cost; morphology control (M); time required (T); scalability (S); and purity (P) of products.

Based on the mechanism and procedure for the fabrication of CP nanohybrids, the methodologies may be classified into three main categories: (i) *ex situ* (sequestered) synthesis, (ii) *in situ* (sequential) synthesis; and (iii) one-pot (concurrent) synthesis [5], as represented in Table 3 and Figure 5. The main characteristic of all the methods in the first category is that the CPs and inorganic species are synthesized separately, and the hybridization is achieved during a subsequent step by simple or more complex blending of two or more components in which interfacial tension between the different components determines the major properties of the resulting nanohybrids. These procedures are simple and usually highly suitable for solution-based processing, which is a milestone for enabling mass production and processing via roll-to-roll printing. Another benefit of these methods is the well-established synthesis procedures because each component is manufactured separately using a known process. As for the hybridization process, there are various valuable strategies available, ranging from very simple methods such as mechanical mixing of the two components, to more sophisticated methods such as ligand exchange or layer-by-layer fabrication procedures [45,46,47]. Finally, physical infiltration of CPs into inorganic nanostructures can also be achieved, resulting in highly ordered nanoarchitectures [48,49]. However, this process is not straightforward because the polymeric guest materials may be prevented from infiltrating the pores of the nanostructured host due to high interfacial tension as a result of physical limitations such as the hydrodynamic radius of the polymer, leading to incomplete pore filling; pore-filling ratios as low as 0.5% have been obtained. Finally, differences in the hydrophobic/hydrophilic nature of the two components can further limit the application of such procedures.

In the second category of methods (*in situ* synthesis), at least one component of the nanohybrid is generated in the presence of its counterpart. These techniques can be further classified on the basis of the component formed *in situ*. It is apparent that nanohybrids obtained by *in situ* methods may have several advantages over their *ex situ*-prepared counterparts because the organic/inorganic interface can be better controlled at the molecular level. Specifically, first, in the case of the electrochemical *in situ* route, heterogeneous components can be incorporated into CPs grown in the form of a film on the electrode surface [56]. One benefit of such an approach is that the structure and properties of the hybridized material can be controlled by changing the critical variables such as deposition time and current density. Although a higher current density leads to faster growth, it is not easy to control, and the polymer may be insufficiently compact. For chemical *in situ* syntheses, formation of the nanohybrid is rather straightforward. Most methods focus on strategies where the CPs are formed in the presence of the inorganic species; however, the opposite is also possible, namely, the inorganic component is synthesized *in situ* within the CP. Wet-chemical co-precipitation is an approach for synthesis of CPs with an oxidant such as KMnO_4_ or FeCl_3_, resulting in hybridized structures [57].

Lastly, the third category, namely one-pot syntheses is probably the simplest (but least controllable) method. These “one-pot” synthesis methods are fundamentally based on *in situ* reactions that form organic/inorganic phases simultaneously in a single stage. Both chemical and electrochemical processes can be applied to this group, but co-electrodeposition is more attractive [55]. The general disadvantage of these methods is the poor control of the size and morphology of the resultant hybridized materials.

## 3. Progress in CP Nanomaterial Synthesis

### 3.1. Recent Advances in the Synthesis of CP Nanomaterials

The most significant trials in liquid-phase polymerization have relied on the utilization of templates for controlling the morphology of CPs on a nanometer scale, where solid templates are an excellent candidate for many applications. For example, Huang´s group proposed a strategy using anion spherical polyelectrolyte brushes (ASPB) with silica cores as templates and dopants for the synthesis of conductive copolymer nanocomposites [58]. The highlights of this route can be summarized as follows: (i) the symmetrical spherical structure of the ASPB can guarantee a spherical morphology with enhanced conductivity, and (ii) charged polymer chains can still be included on the periphery of the nanocomposites via control of the length of the brushes and the thickness of the CP shells to improve the stability of the suspension of the nanoparticles. The poly(styrene sulfonate) (PSS) chains on the silica core surfaces not only facilitate uniform deposition of the conducting copolymer poly(aniline-*co*-pyrrole) through electrostatic interaction, they also serve as anionic dopants for the copolymer. Thus, a high concentration of monomers is mainly distributed close to the cores of the ASPB. Similarly, Zhang *et al.* [59] used TiO_2_ nanotube-PSS as a template and dopant for the synthesis of PPy. The advantages of using TiO_2_-PSS are similar to those of using ASPB because it too exhibits the ability to capture and control the ions within the layer of PSS chains via electrostatic interaction. Then, pyrrole monomers are oxidized by ferric ions in the shell of the PSS to generate PPy.

**Figure 6:**
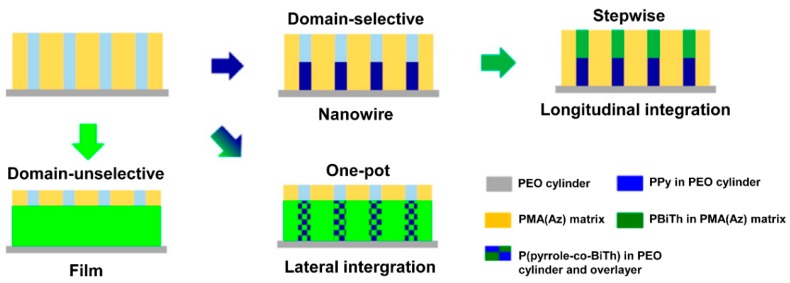
Comparison of stepwise (above) and one-pot (below) electropolymerization using PEO*_m_*-*b*-PMA(Az)*_n_* (polymethacrylate with azobenzene-mesogen in side chain )block copolymer as a template. Reprinted with permission from [60]. Copyright 2015, American Chemical Society.

Block copolymers can also be used as templates, where CPs are generally synthesized in a microphase-separated domain. For example, Komiyama *et al.* [60] reported electropolymerization of pyrrole and bithiophene (BiTh) using PEO-filled cylindrical domains of a microphase-separated PEO*_m_*-*b*-PMA(Az)*_n_* as a template, where the PMA(Az) indicates polymethacrylate with azobenzene-mesogen in side chain. The results were very impressive: while one-pot polymerization gave lateral-composition-modulated copolymer structures, stepwise polymerization created PPy-PEDOT or PEDOT-PPy nanowire heterojunctions (Figure 6).

As a possible milestone for industrial fabrication of colloidal crystal templates, Choi *et al.* [61] demonstrated an efficient method to fabricate large-area two-dimensional (2D) colloidal crystals as a template for nanopatterning of CPs; this method involves the electrophoretic deposition of negatively charged polystyrene colloidal particles and the assembly of the colloidal particles to 2D colloidal crystals at the air-water interface. Similarly, other solid template methods have been developed, such as nanosphere lithography-mediated electrodeposition [62,63], in which Langmuir-Blodgett deposition and drop casting were used to fabricate nanosphere templates.

Although the nanostructural fabrication of CPs has been achieved with nanoporous solid templates via electropolymerization, this process is still challenging. In particular, templates with high-aspect-ratio nanopores suffer from poor monomer diffusion in liquid-phase polymerization. A solution for this problem might be the use of supercritical fluids such as supercritical trifluoromethane (scCHF_3_), which has a relatively high solubility and dielectric constant without the need for additives (e.g., polar solvents) [64,65]. Supercritical fluids are characterized by lower viscosities and large diffusivities as compared to conventional liquids because the physicochemical properties of supercritical fluids are intermediate between those of liquids and gases.

**Figure 7:**
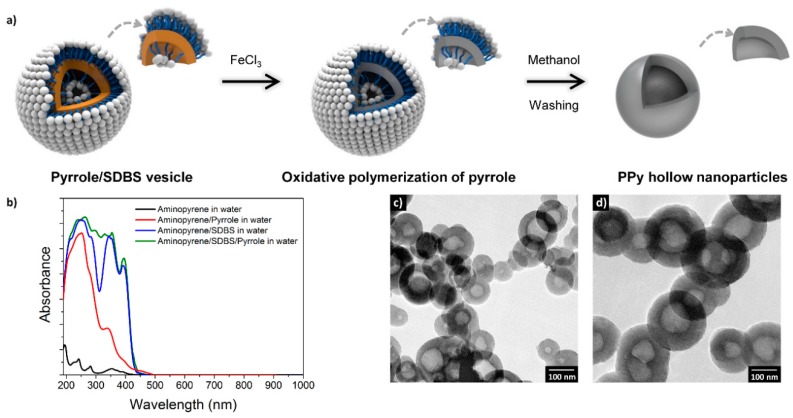
Preparation of PPy hollow nanoparticles. (**a**) Schematic of the formation mechanism, (**b**) UV-vis absorption spectra of aminopyrene in different solvents; (**c**,**d**) Transmission electron microscopy (TEM) images of the hollow PPy nanoparticles with diameters of 100 and 200 nm, respectively. Reprinted with permission from [67]. Copyright 2015, Nature Publishing Group.

Molecular templating has also continued to play a key role in CP synthesis. Note that molecular templating is not only used to control the morphology of the resultant nanoparticles [66], it also affects the doping state of the CPs, which determines the electrical properties [67]. A recent notable example of such a formation mechanism is outlined in Figure 7. PPy hollow nanoparticles with different diameters were prepared by surfactant templating and their electrical properties depended on the size. Devaki *et al.* [68,69] explored liquid crystalline templates with an inexpensive and abundantly available industrial by-product, the main constituent of cashew nut shell extract, namely, cardanol(3-pentadecylphenol). This approach might be a good alternative to traditional petroleum-based surfactants, which have become expensive owing to increased demand and depletion of resources. Acid derivatives of cardanol, including 3-pentadecyl phenol-4-sulfonic acid and 3-pentadecylphenyl phosphoric acid, have a unique amphiphilic design, with the acid group as a hydrophilic head group and a long alkyl chain as a hydrophobic tail. Importantly, the monomer 3,4-ethylenedioxythiophene (EDOT) formed liquid crystalline templates of various shapes with the acid derivative of cardanol, such as discs, gyroids, columns, and lamella.

Much effort has been invested in finding the best composition for use as a molecular template. For instance, Dutt *et al.* [36] fabricated swollen liquid crystalline templates with a quaternary mixture containing brine, SDS as a surfactant, 1-pentanol as a co-surfactant, and cyclohexane oil. In addition, insoluble complex templates with lamellar mesostructures were formed with a noble metal compound and a cationic surfactant such as CTAB [70]. Other surfactants that have been investigated include indigo carmine [71] and Pluronic^®^ F127 [72].

By combining templates, including solid and molecular templates, many interesting results have been reported. Sharma and co-workers [73] employed monodispersed poly(methyl methacrylate) (PMMA) colloids ~430 nm in diameter as three-dimensional (3D) colloidal crystal templates and SDBS as a surfactant for fabricating 3D macroporous PPy inverse opals. There are three main advantages to this method: (i) although PMMA colloids are solid templates and require chemical treatment to remove them, they can be easily dissolved by common solvents, (ii) the inverse opal PPy presented a higher surface area (19.2 m^2^·g^−1^) as compared to conventional non-porous PPy films (4.8 m^2^·g^−1^); and (iii) SDBS not only acts as a surfactant but also a dopant for PPy to trap cations present in the surrounding solution. Other new developments for combining templating methods have also been reported. Rivero *et al.* [74] designed a new approach using mesostructured SiO_2_ as a solid template to synthesize self-doping copolymer poly(aniline-*co*-aminobenzoic acid) in the presence of CTAB. When CTAB was added to the synthesis medium, the silica template showed well-ordered growth with parallel channels approximately 2–3 nm in width. On the other hand, in the CTAB-free precursor solution, a microporous silica deposit was obtained. It should be noted that the redox current response of the as-prepared film appeared only after the surfactant molecules were removed [75]. Moreover, the effect of the time when the anionic surfactant SDS was added during the template polymerization of the CPs was also investigated [76].

Using a method based on template-free electrochemical polymerization, Liao *et al.* [77] successfully fabricated PPy nanostructures of several different morphologies on a prenucleated PPy thin film and examined their growth mechanism using 3D AFM imaging and field-emission scanning electron microscopy (FE-SEM). Interestingly, β-naphthalenesulfonic acid used as a dopant led to differences in the self-assembly behavior of further growing PPy chains at different redox states. These differences determined the final morphology of the PPy nanostructure.

Various impressive approaches have also been developed, such as direct growth by chemical micropatterning [78], bipolar electrochemistry [79,80], enzymatic polymerization using sodium bis(2-ethylhexyl) sulfosuccinate (AOT) as a template [81], and edge nanoimprint lithography [82]. However, due to their complexity, such methods are not popular for the synthesis of CP nanomaterials for applications, and they will require significant time for improvement. Table 4 lists representative examples of synthesis methods for CP nanomaterials developed over the last three years.

In addition to the development of templating techniques to synthesize CP nanomaterials, the investigation of various crucial processing factors has been undertaken. For the chemical route, these include oxidant/monomer ratio and washing post-treatment [9], the chemical effects of the solvent (solvent-free *versus* solvent-limited syntheses) [10], effects of the dopant (d-tartaric acid [11], aromatic and amphiphilic sulfonate [17]), and the polymerization kinetics [94,95]. For the electrochemical approach, investigations of the deposition potential and temperature [96] and chloroform as the environment [97] have been undertaken. Finally, a new anionic dopant, sulfanilic acid azochromotrop [18], was investigated for a combination of chemical and electrochemical polymerization [13,14]. Based on the electrochemical route, the electrophoretic force was successfully exploited for the deposition and assembly of CP nanomaterials. As compared to other approaches, electrophoresis offers advantages such as additional controllable synthetic parameters, high deposition rate, and large-area processing, factors that are essential for industrial manufacturing. Although good results have been achieved for doped CPs patterned onto microscale templates by electrophoresis, the dimensions of the assembled CPs were limited to the micron scale. In order to solve this problem, Shen *et al.* [98] made PANI directly assembled onto multiscale sub-micron templates using a dielectrophoretic technique. In electrophoretic assembly, by applying a uniform electric field, charged “particles” are deposited onto electrodes in a solution, whereas with dielectrophoretic assembly, the “particles” are subjected to a non-uniform electric field and thus need not be charged. The dielectrophoresis approach has advantages over electrophoresis, such as avoiding oxidation of the template surface, meaning that the template can be reused and the materials can be assembled without charging. Consequently, PANI was successfully deposited uniformly onto 100-nm-wide electrodes with multiple length scales.

**Figure 8:**
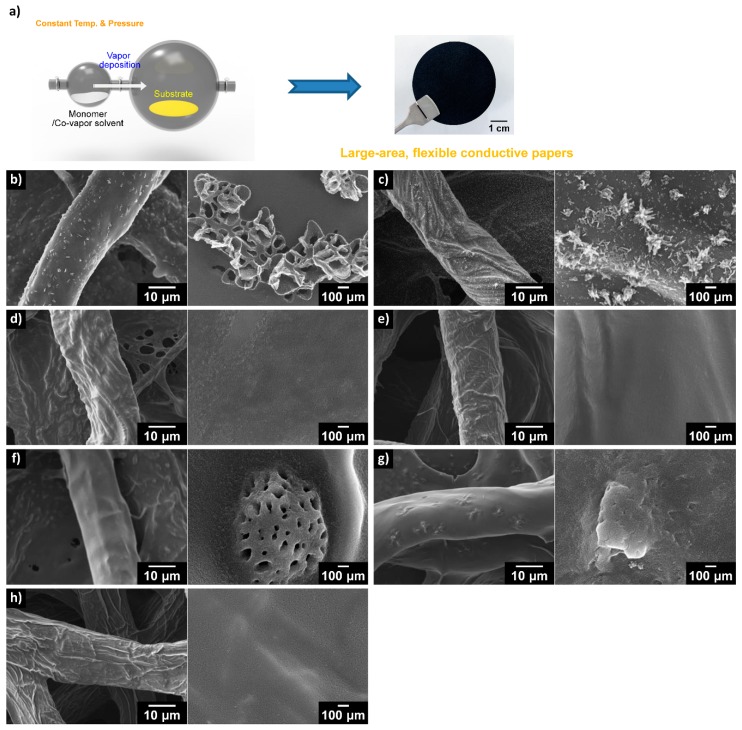
(**a**) Vapor-phase polymerization (VPP) of pyrrole with co-vapors on a cellulose substrate and a typical photograph of as-prepared paper; (**b**–**h**) Scanning electron microscopy (SEM) images of papers prepared with co-vapors: (**b**) methanol, (**c**) ethanol; (**d**) water; (**e**) hexane; (**f**) toluene; (**g**) benzene; and (**h**) no vapors. (left: low magnification, right: high magnification). Reprinted with permission from [105]. Copyright 2015, Nature Publishing Group.

For non-liquid phase polymerization, the two main routes are solid phase and vapor phase (Table 5). Solid-phase polymerization is rarely used for CP synthesis, although it is very simple. Bhandari *et al.* [99] presented the first study of PANI nanowires prepared using a mechanochemical route; however, to date, the advantages of such a method are not clear. Meanwhile, the two methods for synthesizing CPs in a vapor phase are quite efficient, namely vapor-phase polymerization (VPP, often called vapor deposition polymerization, VDP) and chemical vapor deposition (CVD). The distinction between these methods is in how the initiator is applied during polymerization. The initiator and monomer must be in a vapor phase in the CVD process, whereas only the monomer needs to be in the vapor phase for VPP. The morphology of CPs using the VPP method is highly affected by the synthesis conditions, as well as by the substrate curvature [100]. As compared to liquid-phase polymerization, CVD and VPP can produce thinner films with better reproducibility and higher crystallinity and conductivity. To date, vapor-phase synthesis has provided unique possibilities in terms of tuning the synthesis process of CPs; even multi-dimensional CP nanostructures can be fabricated via this route [101]. The latest work reported that co-vapors can be introduced into the VPP process to modulate the morphology and properties of the resulting CP product (Figure 8) [82]. It was found that co-vapors such as alcohols and water can affect (i) the vaporization of the monomer, (ii) the orientation of forming polymer chains; (iii) the rate and efficiency of oxidative polymerization; (iv) the swelling of the substrate; and (v) the diffusion of the monomer into the substrate, all of which lead to significant changes in the characteristics of the resultant CP.

### 3.2. Novel Trends for Synthesis of CP Nanohybrids

Recently, nanohybrids have fascinated many researchers because of their usefulness in many applications. The fabrication of these hybrids can be commonly achieved via the combination of CPs and inorganic species in the nanometer regime, as discussed above. A notable example is manganese oxide (MnO*_x_*)/CP nanohybrids [51,67,111,112]. Because MnO*_x_* can act as a reactive template in an acidic medium for the chemical polymerization of CP, direct CP nanocoating has been made on the surface of MnO*_x_*. The resulting nanohybrids with an optimized ratio of MnO*_x_*/CP showed the following merits: (i) the coated CP provided conductive pathways for better utilization of MnO*_x_*, and (ii) both MnO*_x_* and CP exhibited synergistic electrochemical properties. It has been noted that the properties of hybrid nanomaterials may vary significantly depending on the preparation methods even for the same materials. Characteristics of the preparation methods can be recognized by comparing the synthesis examples of MnO_2_/PANI using adsorption templates [113] and reactive templates [114,115]. Interestingly, in electrochemical capacitor applications, the results using the adsorption template method showed higher specific capacitances than their counterparts using the reactive template method. As a partial summary, Table 6 shows several representative examples for nanohybrid synthesis using CPs and metal- or metal oxide-based species.

In general, a high-surface-area composite with high conductivity is desired to achieve superior performance for applications of CPs. Therefore, in addition to the optimization of the main properties such as electrical conductivity, proper control of the corresponding morphology (such as pore size and specific surface area) is critical. Using simple thermal treatments and chemical VPP, Yang *et al.* [111] successfully synthesized MnO_2_ nanoparticles/PEDOT porous hybrids. The highlight of this porous hybrid is an “open” porosity, resulting in excellent electrochemical activity due to the synergistic properties between the nanoparticles and CPs, even though the electrical conductivity decreased with increasing nanoparticle content.

One of the most well-known routes for *in situ* synthesis of CPs within a nanostructured host metal oxide is the infiltration method. The simplest approach is physical infiltration; however, such infiltration is often hindered due to the high interfacial tension between the host and the guest. In addition, the combination of the physical infiltration approach and electrochemical polymerization requires positive potentials, which can result in irreversible oxidation of the CPs. Using photoelectrochemical polymerization, Samu *et al.* [75] overcame this problem. As a first step, an EDOT monomer is oxidized by photogenerated holes on the surface of a metal oxide. The polymerization is initiated by sensitizer nanoparticles. Next, the electrochemical growth of PEDOT starts to take place predominantly on the surfaces of the nanoparticles. Mazzotta *et al.* [30] also successfully demonstrated that highly flexible CP microstructures can be achieved from a silicon template via light-activated electrochemical processes. A summary of recent photosynthesis studies for CP nanohybrids reported in the literature is given in Table 7.

Carbon and carbon derivatives such as carbon nanotubes (CNTs), graphene, graphene oxide (GO), and reduced graphene oxide (rGO) are other potential components for the synthesis of nanohybrids (Table 8). Ansari *et al.* [32] synthesized graphene/PANI nanocomposites using an *in situ* oxidative polymerization in the presence of CTAB as a surfactant. Graphene nanosheets were uniformly distributed in the PANI matrix with the aid of CTAB, leading to high conductivity and thermal stability via the π–π interactions between graphene and PANI. Notably, *p*-toluenesulfonic acid used as a dopant also contributed the enhanced electrical and thermal properties. Similarly, it is noteworthy that several dopants, such as citric acid [143], 1,5-naphthalene disulfonate, 2-naphthalene sulfonate [144], amaranth, and pyrocatechol violet [145], influence the main structure and properties of nanohybrids.

Many studies have focused on the use of GO or rGO with CPs. For instance, Wang *et al.* [146] synthesized nanohybrid electrode materials through *in situ* polymerization of thiophene-grafted GO in the presence of an EDOT monomer and investigated the covalent linking of GO with PEDOT chains. Via the covalent coupling of GO with the 3-position of the thiophene unit, the promotion of the coupling of the EDOT monomer in the 2-, 5-positions of the thiophene unit reduced undesirable coupling.

**Figure 9:**
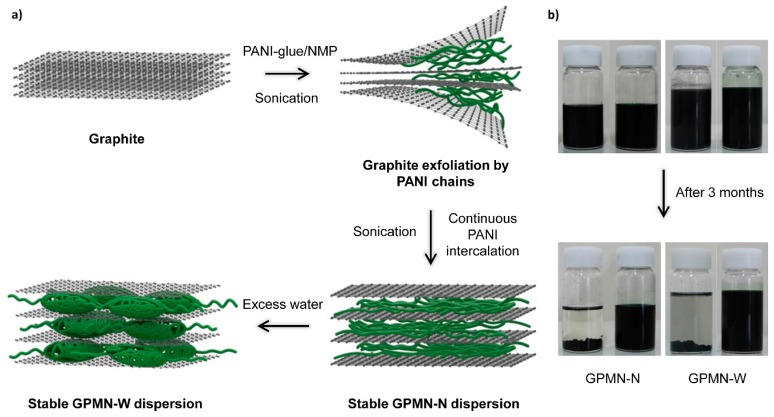
(**a**) Schematic illustration of the formation of graphene/PANI multilayered nanostructures (GPMNs) by direct physical exfoliation of graphite with PANI glue; (**b**) Photographs showing the long-term colloidal stability of the GPMN dispersion solution in the absence (left) and the presence (right) of PANI glue. GPMNs showed outstanding colloidal stability in both *N*-methyl-2-pyrrolidone (NMP) and water. Reprinted with permission from [45]. Copyright 2015, John Wiley & Sons.

Exfoliation of graphite by CPs may be an efficient strategy for functionalization of graphene with CPs [147]. This method is more beneficial than the conventional approach due to the high electrical conductivity of pure graphene layers. As can be seen in Figure 9, a notable recent work reported the fabrication of free-standing flexible graphene/PANI multilayered nanostructure (GPMN) films via simple physical intercalation of CP into graphite [45]. The resulting nanohybrids not only showed excellent electrochemical performance, they also exhibited high dispersion stability in *N*-methyl-2-pyrrolidone (NMP) and water for more than three months at room temperature.

PEDOT:PSS is a mixture of PEDOT and PSS polymers that provides good processability, such that aqueous dispersions of PEDOT:PSS are commercially available. The coupling of PEDOT:PSS with nickel hydroxide Ni(OH)_2_ and multi-walled carbon nanotubes (MWCNTs) has also been examined [161]. In this ternary architecture design, various synergistic effects were observed in their application as the electrode material for supercapacitors. First, MWCNTs with relatively low cost, large specific surface areas, and excellent conductivity serve as an ideal conductive template for Ni(OH)_2_ deposition. Second, instead of crystalline Ni(OH)_2_, which is commonly used as a pseudocapacitive material, amorphous Ni(OH)_2_ was chosen because structural disorders in Ni(OH)_2_ can greatly improve the electrochemical efficiency. Finally, wrapping the MWCNT/Ni(OH)_2_ with PEDOT:PSS significantly reduced the contact resistance among the metal oxide/hydroxide-deposited MWCNTs. Another interesting result of this ternary system was proposed by Ramasamy *et al.* [162], where an effective and simple route for fabricating a ternary nanohybrid via the combination *in situ* polymerization and electrodeposition method was presented. These examples share similar attributes in the sense that an external component (either a metal-based semiconductor, a nano-carbon, or CPs) is introduced to assist the fabrication of the ternary CP nanohybrids. A partial summary of recent research is shown in Table 9; there are a wealth of examples illustrating the fabrication of ternary hybrids.

**Figure 10:**
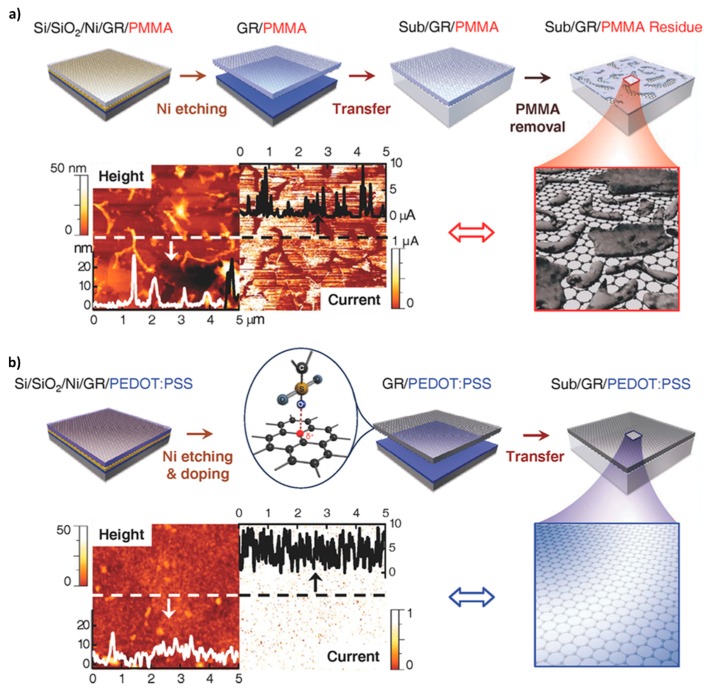
Schematic illustrations and representative conductive AFM analysis for (**a**) conventional transfer and (**b**) “doping transfer” methods. PMMA and PEDOT:PSS were used as the support films in both methods. Reprinted with permission from [183]. Copyright 2014, John Wiley & Sons Inc.

Interestingly, PEDOT:PSS has been employed as a dopant for graphene films. For example, Lee *et al.* [183] developed the so-called “doping transfer” approach, providing a unique solution to the residue problem of the PMMA-assisted method in graphene substrate transfer (Figure 10). There were two interesting points reported. First, the addition of a PEDOT:PSS layer decreased the optical transmittance value by only 1%. This was attributed to the following effects: (i) the strong cohesion between PEDOT:PSS and the graphene film due to the strong attraction between the sulfonic acid group of PSS and graphene, and (ii) the increased cohesion within the PEDOT:PSS layer, which was ascribed to the cross-linking effect of the PSS chains during processing. Second, the doping effect of PEDOT:PSS on graphene can be accomplished via the following three mechanisms: (i) the attraction of electrons from the graphene to the PSS chains, (ii) charge-transfer doping due to electron transfer from graphene to PEDOT:PSS by a difference in the work function; and (iii) percolation doping, which bypasses the charge through the conductive film across resistive grain boundaries in the graphene. Furthermore, doping stability was also achieved through the environmental stability of PEDOT:PSS and the chemical robustness of graphene. Finally, the benefits of the simplified process, the enhanced purity, and the stable doping effect made it possible to generate reliable and versatile graphene/CP nanohybrids.

## 4. Selected Applications of CP Nanomaterials

Polymers have advantages over metals or inorganic semiconductors, such as easy synthesis and processing, chemical and structural diversity, low weight, and flexibility. CPs have provided further advantages arising from the inherent π-electron-conjugated system, which has made it possible to develop many competitive applications in various fields, such as sensors, energy conversion/storage devices, and drug carriers.

### 4.1. Sensors

A great deal of effort has been made to develop various types of CP-based sensors [4,6,8,101,184,185,186]. The signal transduction mechanism of CPs for sensor applications has mostly relied on changes in the electrical properties. Recently, there has been an effort to use multiple signal transduction mechanisms to obtain more accurate information on the target species. Zhong *et al.* [186] designed a gas sensor based on conductive opal photonic crystal films. CPs were deposited into the void spaces of silica colloidal crystal templates. The subsequent silica etching resulted in an inverse opal structure. Upon exposure to ammonia gas, both electrical and optical signals were successfully monitored using the conductive photonic crystal film, which allowed for enhanced response time and accuracy.

**Figure 11:**
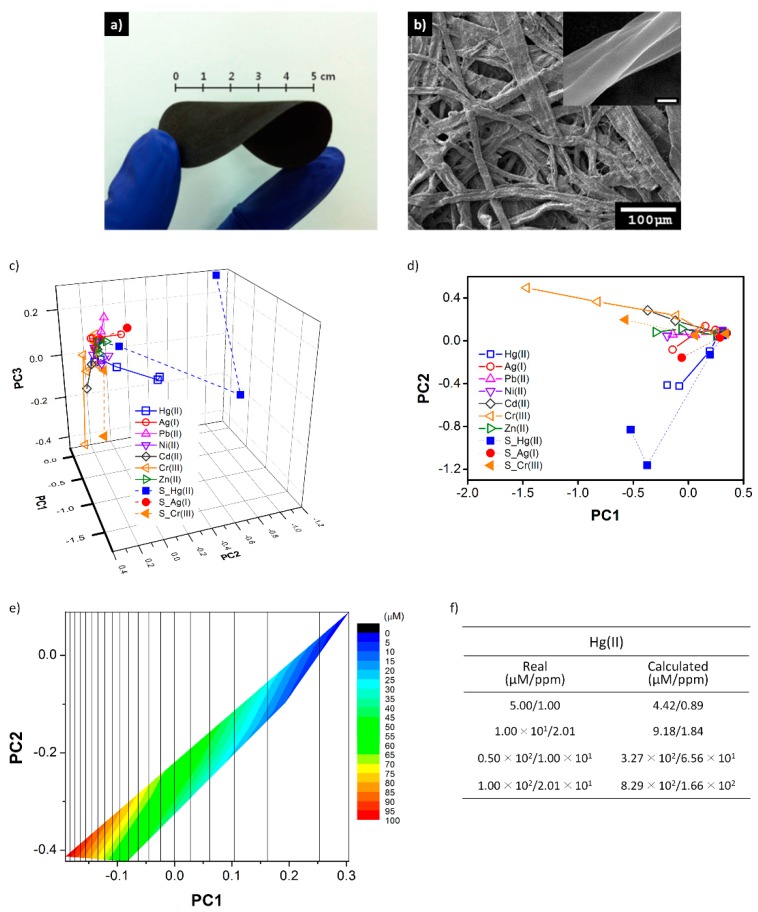
Photograph showing (**a**) the flexibility of a large-area, freestanding PPy/cellulose paper; (**b**) SEM images of a PPy/cellulose paper (inset: high-magnification image, the scale bar is 200 nm); (**c**,**d**) Principal component analysis plots of the responses of PPy/cellulose papers with data from real samples; (**e**,**f**) Calculation of Hg(II) concentration in groundwater. Reprinted with permission from [187]. Copyright 2014, Royal Society of Chemistry.

Another critical issue in CP sensors is that it is very difficult for CP sensors to achieve high selectivity for specific target species. Commonly, many data are collected from a sensor array, and then statistical tools such as principal component analysis for data processing are utilized to evaluate the selectivity of the sensor. This approach was well demonstrated by a recent study using PPy-coated cellulose papers as the sensing material (Figure 11) [187]. PPy/cellulose papers with different oxidation levels were prepared at different applied potentials, and their ability to electrochemically recognize metal ions was systematically investigated. Unique signal patterns were observed for Hg(II), Ag(I), and Cr(III) through principal component analysis of the response dataset, which support the potential for using the PPy/cellulose paper as a high-selectivity sensor for detecting the three metal ions.

It is also challenging to develop simple, inexpensive, and large-area processing techniques for fabricating nanostructured sensor devices. Electrodeposition can be a good candidate technique to satisfy those requirements. For example, Ferrala *et al.* [188] achieved the rapid electrophoretic assembly of nanostructured PANI on a gap between microelectrodes by applying an alternating current electric field. A significant benefit is the short assembly time (approximate 5–10 s), despite the fact that electrode gaps are micrometer-sized. The combination of forces such as dielectrophoresis, alternate current electro-osmotic flow, and induced-charge electrokinetic flow, speeded up the assembly process. Consequently, the nanostructured PANI-based sensors exhibited excellent reversibility to low concentrations (a few ppm) of ammonia gas at room temperature, even after storage in air, as well as high sensitivity.

**Figure 12:**
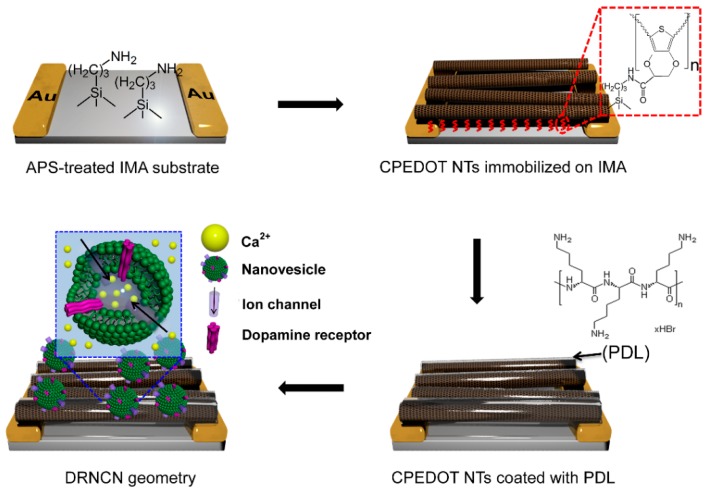
Schematic illustrations of construction steps for receptor geometry, where IMA, CPEDOT NTs, PDL, and DRNCN note interdigitated microelectrode array, carboxylated PEDOT nanotubes, poly-d-lysine, and dopamine receptor-containing nanovesicles-immobilized CPEDOT NTs, respectively. Reprinted with permission from [184]. Copyrights 2014, Nature Publishing Group.

Remarkably, the field-effect transistor (FET) configuration has been used to achieve enhanced sensitivity. Receptor-modified CP nanomaterials are deposited as a channel bridging the source and drain electrodes. When the receptor recognizes a target species, the gate potential being applied on the channel changes, which in turn modulates the source-drain current. With this operating mechanism, many different types of FET sensors have been developed to detect glucose [189], odorants [190], proteins [191,192], and hormones [193]. Recently, a high-performance dopamine FET sensor was successfully demonstrated using human dopamine receptor-containing nanovesicles as the gate-potential modulator on a PEDOT nanotube channel (Figure 12) [184]. The lowest detection level was as low as 10 pM, which was 10 times more sensitive than that of previously reported CP-based dopamine sensors.

Surface acoustic wave (SAW)-based sensors are also an interesting approach for gas detection due to their many benefits, such as small size, high sensitivity, and fast and reliable response. Li and coworker [40] spin-coated PPy nanoparticles onto SAW transducers to trace acetone gas. The nanoparticle layer coated onto the ZnO intermediate layer showed rapid response/recovery times and excellent sensitivity resulting from low gas-diffusion resistance. Additionally, the responses of the sensors were linearly proportional to the acetone concentration in the range from a few to a few tens of ppm.

Among various CPs, application of nanostructured PANIs as sensing materials has been widely explored. Chiam *et al.* [194] prepared a colorimetric alcohol sensor using PANI-coated glass microfibers with laser light source with a wavelength of 1550 nm. A redshift in the output spectrum was observed via electrostatic interaction between the partial positive amine group of PANI and the partial negative hydroxyl group of alcohol.

### 4.2. Electrochemical Energy Storage Devices

**Figure 13:**
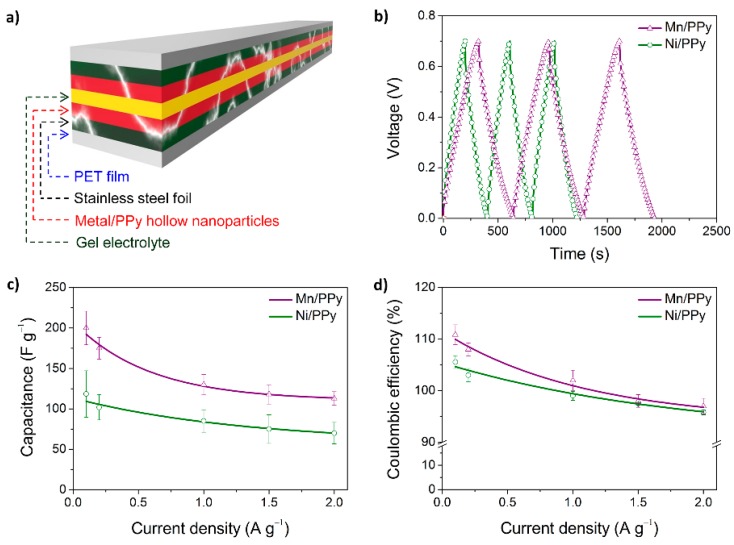
Flexible, all-solid-state cells based on Mn/PPy hollow nanoparticle electrodes. (**a**) Pictorial representation of an all-solid-state flexible electrochemical capacitor cell; (**b**) typical galvanostatic charge/discharge curves of metal/PPy hollow nanoparticles measured at a current density of 0.1 A·g^−1^; (**c**) specific capacitances measured at different current densities; and (**d**) calculated coulombic efficiencies. Reprinted with permission from [67]. Copyright 2015, Nature Publishing Group.

There is an increasing demand for high-performance energy storage devices. A variety of electrode materials have been designed and tested so far, including CP nanomaterials. As a recent remarkable example, PPy hollow nanoparticles were simply prepared using surfactant templates and their performance as the electrode for electrochemical capacitors was examined (Figure 13) [67]. The hollow nanoparticles served as a nanocage to prevent metal ion leaching during charge/discharge, thus allowing excellent capacitance retention. In addition, pseudocapacitive metal species (e.g., Mn- and Ni-related species) were readily deposited on the inner and outer surfaces of the hollow nanoparticles, providing enhanced capacitances.

**Figure 14:**
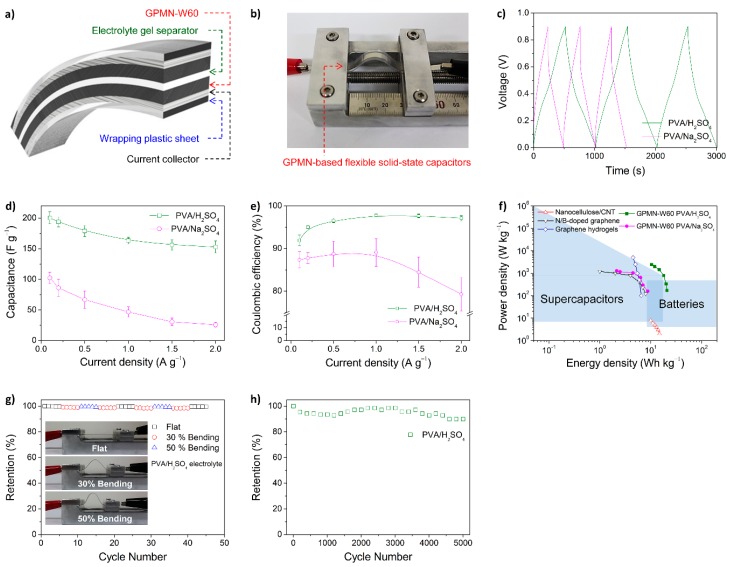
Flexible, all-solid-state graphene/PANI-glue multilayered nanostructure cells: (**a**) schematic of an all-solid-state flexible electrochemical capacitor cell; (**b**) typical photograph of cell performance measurement under mechanical deformation; (**c**) galvanostatic charge–discharge curves at a current density of 0.1 A·g^−1^ for different electrolytes; (**d**) specific capacitances as a function of current density; (**e**) coulombic efficiency as a function of current density; (**f**) Ragone plots; (**g**) capacitance as a function of bend radii; and (**h**) long-term cycling stability of the cell with an acidic electrolyte. Reprinted with permission from [45]. Copyright 2015, John Wiley & Sons Inc.

It should be noted that control over the microstructure and properties of a material does not guarantee improvement in the material’s performance because each material has an intrinsically limited capacitance. Therefore, different kinds of materials have been hybridized to obtain desirable synergistic effects. Typically, graphene/CP nanohybrid electrodes can be used to fabricate high-performance, flexible, solid-state electrochemical capacitors. Very recently, PPy nanospheres were intercalated into stacked graphene layers, which resulted in a unique 3D opened structure that allowed facile electrolyte diffusion [147]. As a result, the nanohybrids had high capacitance, as well as good long-term cycling stability. In addition, importantly, the packing density of the nanohybrids varied with the nanosphere content, indicating the potential for high volumetric capacitance. Similarly, the fabrication of graphene/PANI multilayered nanostructures has been reported [45], in which graphite was physically exfoliated by PANI glue (Figure 14). The physically exfoliated graphene provided much better electrical properties than GO and rGO. The graphene/PANI nanostructure-based electrode showed a high specific capacitance of 200 F·g^−1^, a cycling stability with capacitance retention of ~80% after 5000 charge/discharge cycles, and excellent flexibility.

In addition to electrode materials, other components such as additives, electrolytes, and separators can be tailored to provide better device performance. Employing redox-active electrolytes can be a good approach toward enhancement of the pseudocapacitive performance of CPs [195]. Prepared by simply adding a redox mediator to the conventional electrolyte, redox-active electrolytes have shown significant improvements in the specific capacitance of pseudocapacitors using various CPs such as PANI, PPy, and PEDOT as compared to conventional electrolytes. In addition, electrode systems such as symmetric/asymmetric electrode configuration and the weight ratio of two electrodes is also a key factor affecting the performance of the capacitor. Our group well demonstrated the importance of the electrode system in electrochemical capacitors using two different nanohybrid electrode materials, namely, MnO_2_/PEDOT nanotubes and rGO/CNFs [196]. Lastly, the latest review article addresses major issues in electrochemical capacitor application using nanostructured electrode materials [197].

### 4.3. Photovoltaic Cells

Dye-sensitized solar cells (DSSCs) have become popular for various energy conversion applications. A typical DSSC consists of a transparent photo-anode with a dye-sensitized mesoporous thin film, a redox couple, and a counter electrode with a catalytic layer deposited on a fluorine-doped tin-oxide (FTO) substrate [17,37,48,142,162,180,198,199,200]. Recently, much attention has been paid to CPs as a relatively low-cost device for photovoltaic application due to their excellent electrocatalytic activity, high electrical conductivity, and good film-forming ability [198,201]. For example, Kung *et al.* [198] examined a PEDOT hollow microflower array film as the catalytic material on the counter electrode of a DSSC. The PEDOT hollow microflower arrays contributed to a high power conversion efficiency of 7.20%, which was comparable to that of a cell with a sputtered Pt film (7.61%). Considering the low value of the Warburg diffusion resistance of the DSSC, it was believed that the unique microflower structure would facilitate a kind of hemispherical diffusion of ions for the electrocatalytic reaction in the electrolyte region. The lower the Warburg diffusion resistance of the DSSC, the higher the fill factor of the cell (a critical parameter for evaluation of the cell performance). Lin *et al.* [180] reported the effect of PEDOT coating on the electrocatalytic activity of CNT/polypropylene plates. Interestingly, it was found that the PEDOT-coated CNT/polypropylene plate showed a Pt-like electrocatalytic activity for I_3_^−^ reduction. Consequently, a DSC with the PEDOT-coated CNT/polypropylene counter electrode showed a high power conversion efficiency of 6.77%, even after the bending test.

From a survey of numerous studies, it can be confirmed that the application of CP nanomaterials as potential substitutes for Pt counter electrodes in DSSCs is a recent trend. Nevertheless, a clear mechanism to describe the function of CP nanomaterials as counter electrode materials still needs to be clarified. It is anticipated that the use of nanostructured CPs will open a new avenue for industrial fabrication of DSSCs due to their low cost and reasonable performance as compared to Pt electrodes [199].

### 4.4. Drug Carriers

Recently, there has been substantial effort to fabricate drug delivery systems based on smart materials [73]. CPs that possess ion-exchange properties are being studied as promising materials for drug reservoirs for this purpose. In contrast to physical entrapment, CPs exhibit reversible electrostatic immobilization. The mechanism of this process relies on the fact that CPs undergo a charging-discharging process and adopt positive/negative charges, which strongly depend on their oxidation state. These charges attract ions of the opposite charge in the polymeric matrix, linking them via coulombic interactions. Hence, CPs can immobilize anionic drugs during oxidation (doping), move to a target site, and release the drug via the process of reduction (dedoping).

In a trial of a drug delivery solution for anticancer treatment, Krukiewicz *et al.* [202] fabricated a robust and low-cost nanocomposite based on PEDOT and oleanolic acid. The materials exhibited competitive characteristics such as good biological activity of the embedded drug and well-controlled release, making it a potential candidate for further development and a promising commercial application. Hong *et al.* [203] proposed an integrated strategy with conductive disulfide–biotin-doped PPy nanowires for cell capture, on-demand release, and *in situ* quantification of captured cells within the same platform. The PPy nanowire platform showed very high sensitivity and specificity. In addition, the enzymatic reduction of hydrogen peroxide on PPy resulted in a greatly increased amperometric response with a wide range and low limit of detection. Interestingly, this helps us to achieve a better understanding of circulating tumor cells, with the aim of unraveling the complex mechanisms governing cancer biology.

**Figure 15:**
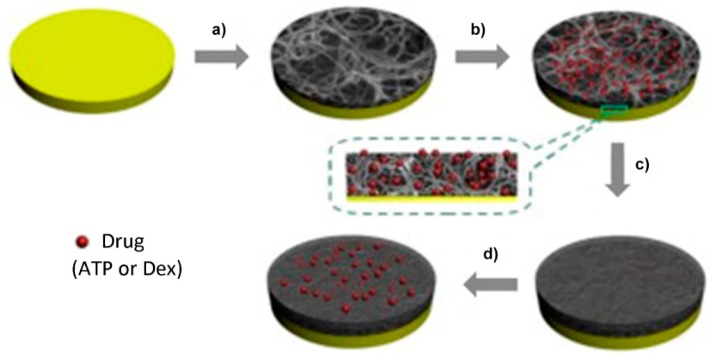
Schematic process showing the fabrication and drug release of the drug-loaded PPy nanowire network coated by a PPy film: (**a**) electropolymerized PPy nanowire network; (**b**) addition of the drug; (**c**) preparation of the PPy film by CVD; (**d**) application of electrical stimulation to release drug. Reprinted with permission from [106]. Copyright 2013, Elsevier.

Although the mechanisms of CP-based drug carriers are very attractive due to the inherent doping–dedoping and stimulus–responsive properties of CPs (where drugs can be incorporated into polymer chains as doping agents and then released through electrical stimulation), the capacity of drug loading is usually low because it is strongly dependent on the doping level. Furthermore, the types of drugs that can be used are limited to charged and small molecules. Therefore, a limited range of drugs and relatively low drug-loading capacities are two main drawbacks for the practical application of CP-based drug delivery systems. In order to partly solve those problems, Jiang *et al.* [106] found that nano- or micro-sized inter-nanowire pores of a PPy nanowire network can serve as reservoirs to store drugs. In addition, it is not necessary to consider the volume and the charge of the loaded drugs because the space for drug storage has been changed from the backbones of the CPs to the vacancies among the nanowires. Consequently, both the range of loaded drugs and the drug-loading capacity can be effectively improved. Herein, a PPy film was further deposited on top of a PPy nanowire network by chemical vapor deposition to inhibit leaking of the stored drugs (Figure 15).

## 5. Conclusions and Outlook

To date, an enormous number of approaches have been developed to fabricate CP nanomaterials. The goal of this review article was to better illustrate the recent advances in the synthesis of CP nanomaterials and their potential applications. CP nanohybrids are also promising candidates because they show interesting properties not observed in the individual components and their bulk counterparts. There is no doubt that the hybridization of CPs on a nanoscale offers a very broad and promising “toolbox” for research, not only to solve the abovementioned challenges and enhance the corresponding properties, but also to achieve multifunctional nanosystems. However, an easy, efficient, and scalable synthesis method for nanostructured CPs with desirable sizes, microstructures, and properties is still in demand. Furthermore, there are still limitations in the application of nanoscale devices, such as reproducibility, stability of the properties of CPs, and control of individual nanostructures.

Specifically, in terms of the major properties, there are several critical challenges in achieving further advances in CP research: (i) metallic conductivity; (ii) processability; and (iii) environmental stability. First, the common explanation of the conductive mechanism of CPs is suggested by the Drude model: charge carriers hop between ordered metallic domains instead of unordered amorphous domains [26,107,204]. Thus, a high degree of structural order would lead to high electrical conductivity. However, there are no effective methods to characterize and control the crystalline/amorphous ratio of CPs, particularly in hybrid systems. This might be the reason why most studies have not focused on the crystalline properties of CPs for achieving metal-like electrical properties. Doping is an essential step for the preparation of CPs. During the last decade, it has been believed that APS and metal salts (e.g., FeCl_3_, Fe_2_(SO_4_)_3_) are the best oxidants; acids such as HCl and H_2_SO_4_ are the common doping agents, in which sulfonic acids such as dodecylbenzenesulfonic acid and camphorsulfonic acid contribute to improvement in the solubility of CPs; and some anionic surfactants are not only molecular templates but also doping agents. More sophisticated doping techniques, such as dual doping, double doping, and triple doping, have also been developed to provide desirable properties and functions to CPs, including metal-like conductivity and water-solubility. However, it should be also noted that the more complex the doping step, the harder it is to control and predict the major characteristics of the resulting CP nanomaterials. In general, CPs are insoluble in common solvents and are not thermoplastic, which has prevented a wide range of application of CPs. As a representative commercialized soluble CP, many researchers have used PEDOT:PSS. Another important issue is that the coupling of PEDOT with PSS enhances the environmental stability of PEDOT’s properties. In fact, PEDOT was initially developed to provide a soluble conducting polymer that lacked the presence of undesired *α*,β- and β,β-coupling of thiophene rings within the polymer backbone [205]. However, the resulting PEDOT was still insoluble, although it fortunately showed stability and high conductivity. PEDOT became soluble in water when blended with a polyelectrolyte PSS. This story about PEDOT:PSS may be a good model for developing processable CPs with stable properties. Of course, combining CPs with PSS cannot be a generalized solution. More efforts should be aimed at designing and synthesizing new monomers and dopants.

Inspired by the Nobel Prize in 2000, numerous researchers have been devoted to CP research. Although the tremendous value of CP nanomaterials is obvious, we believe that their full potential is yet to be realized. Facilitating interdisciplinary research will provide new opportunities for CP nanomaterials, and the intelligent integration of the numerous advantages of CPs with other materials has been and will continue to be a key topic in various applications. It is highly anticipated that further advances in CPs will lead to new horizons in material technology and its application.

## Figures and Tables

**Table 1:** Polymerization routes employed to obtain conducting polymers (CPs).

Route	Details	Ref.
Chemical polymerization	Requires an oxidizing agent to synthesize the polymer. The morphology of the polymer can be controlled by varying the parameters of the process, such as monomer/oxidizing agent concentration, temperature, pH, and reaction time.	[9,10,11,12,13,14]
Electrochemical polymerization	An oxidizing agent is not required for this route, which is an efficient approach for depositing CPs on substrates. Some monomers are theoretically not electropolymerizable. Furthermore, it is difficult to scale up this process. A high oxidation potential may lead to over oxidation of the polymer.	[13,15,16,17,18,19]
Photo-polymerization	Illumination is needed for polymerization. This route was developed to solve the over oxidation problem of the electrochemical method. The process can be well controlled simply by turning the light on or off.	[20,21,22,23,24]

**Table 2:** Synthesis methods of CP nanomaterials.

Synthesis methods	Advantages	Disadvantages
Solid template	Applicable to almost all CPs. Possible to precisely control the size and morphology.	A post-synthesis process is required to remove the template. Nanostructure quantity is confined by the size of the template membrane.
Molecular template	Relatively simple, and thus scale-up is possible under optimized conditions.	Hard to provide good uniformity of size and morphology.
Template-free	Simple process without templates.	Limited to certain precursors.
Electrospinning	Simple to produce continuous CP nanofibers.	Only soluble and thermoplastic polymers are applicable.
Nanoimprinting	High throughput and high resolution.	An expensive micro-mold is required.

**Table 3:** Preparation methods for synthesizing CP nanohybrids.

Main Group	Advantage	Disadvantage	Example	Ref.
*Ex situ* synthesis	Simple Solution-processable	Limited applications Poor control of the contact between each component	Mechanical mixing	[45]
			Layer-by-layer deposition	[46]
*In situ* synthesis	Variable methods based on chemical or electrochemical route. Facile control of many variables	Higher complexity as many parameters need to be considered	*In situ* polymerization. Electrodeposition. *In situ* reduction	[50,51,52]
One-pot synthesis	Simple Short processing time	Limited control over structure and morphology of the products	Redox reaction Co-deposition	[53,54,55]

**Table 4:** Representative examples of fabrication methods for CP nanomaterials developed over the last three years.

CP	Polymerization method	Details	Refs.
PANI	Amyloid nanofiber template polymerization	Amyloid nanofibers were successfully used as templates for the formation of conductive core-shell nanowires	[83]
	Planar DNA template	Production of CPs with controlled shapes on 2D polyelectrolyte templates was investigated for the first time	[84]
	Electrospinning using poly(amic acid) fiber as a template	Hollow nanofibers with controllable wall thicknesses were successfully obtained	[85]
	Dedoped chemical polymerization	Water-dispersed CP nanofibers with high capacitance were achieved by double doping	[12]
	Biphase interfacial polymerization	The mechanism for self-assembly in crystalline 1D nanostructures was investigated	[86]
	Surface-initiated polymerization	A new approach for multimodal core-shell nanoparticles with a stable doping state was reported	[87]
	Interfacial polymerization	A novel hollow PANI nanocapsule with holes in the wall was synthesized	[88]
PPy	Time-dependent template-assisted polymerization	A new synthesis approach for the precise control of wall morphologies of colloidal microparticles was studied	[89]
	Modified pulse potentiostatic method	A good method to control the shape of micelles at the substrate/electrolyte interface and control the morphology of CPs was proposed	[90]
PEDOT	Galvanostatic electrodeposition	Good result combining a carboxylated polystyrene template made by nanosphere lithography with SDS as a molecular template was achieved	[91]
	Non-spontaneous emulsification	A novel method using colloidal chemistry to fabricate multifunctional CPs was developed	[92]
	Electron pulse-enabled *in situ* polymerization	The mechanism of CP growth was investigated experimentally and via modeling	[93]

**Table 5:** Representative reports for the solid/vapor-phase polymerization of CP nanomaterials.

Phase	CP	Details	Refs.
Solid phase	PANI	Hairy CP nanowires were obtained via mechanochemical polymerization using citric acid as a dopant	[99]
	PEDOT	The role of temperature in the solid-state synthesis was studied	[102]
		A low-cost, low-temperature method to fabricate high-performance CPs was successfully developed	[103]
Vapor phase	PANI	The effect of microwave radiation was studied	[104]
	PPy	The role of co-vapor in the vapor-phase polymerization (VPP) method was studied	[105]
		An application for drug storage was carried out by depositing PPy using the chemical vapor deposition (CVD) method	[106]
	PEDOT	The dependence of electrical conductivity on VPP temperature was discussed	[107]
		Single-crystal CP nanowires were developed using VPP with liquid-bridge-mediated nanotransfer printing	[108]
		A one-step fabrication of 2D nanoparticles was investigated	[109]
		The advantages of directly depositing CP nanofibers was demonstrated	[110]

**Table 6:** Representative examples of the incorporation of inorganic species into CPs.

CP	Inorganic species	Preparation method	Details	Refs.
PANI	Au	Interfacial polymerization	The formation mechanism of Au-PANI was presented	[116]
	Cu	Concurrent synthesis	The structure of a Cu/PANI hybrid was studied	[117]
	Pd	Layer-by-layer technique	A Pd/PANI/Pd sandwich-structure nanotube array was first reported	[46]
	TiO_2_	Combination of hydrothermal and electropolymerization	A multicolor electrochromic film was fabricated based on hybrid core-shell nanorod arrays	[118]
	CdO	Chemical oxidative polymerization	Aqueous diethylene glycol solution medium was used for the first time	[119]
	MoS_2_	Vertically aligned chemical polymerization	A good example of hybrid 3D tubular structures was discussed	[120]
	Se_0.5_Te_0.5_	Lyotropic liquid crystalline template	A mesoporous dual-layer film was synthesized using Brij56 surfactant	[121]
	MnFe_2_O_4_	Incorporative polymerization	Dual nature of hybrid (cathode catalyst and anode modifier) was first demonstrated	[122]
PPy	Ag	Incipient network conformal growth technology	A new porous material, namely, an “aero-sponge,” was proposed	[123]
		Cathodic co-deposition	Highly stable sensing activity of the hybrid was studied	[124]
	MnO_2_	Electropolymerization	Effect of deposition time was reported	[51]
	ZnO	Ultrasound-assisted chemical polymerization	Well-controlled granular and layered nanocomposite was formed	[125]
	CuO	Wire template technique	The previous method was extended to study polymerization time	[126]
	CoO	Modified hydrothermal and post-annealing process	3D growth of well-aligned nanowire array was developed	[127]
	MoS_2_	*In situ* intercalative polymerization	A facile strategy for intercalation of PPy into MoS_2_ nanosheets was proposed	[128]
	TiO_2_	Pulsed-light and pulsed-potential method	Good control of the deposition rate was demonstrated	[129]
	LiV_3_O_8_	Low-temperature *in situ* polymerization	A new anode material for rechargeable lithium batteries was reported	[130]
	ZnCo_2_O_4_	Reflux method and chemical polymerization	A facile method for fabricating mesoporous ZnCo_2_O_4_-coated PPy was developed	[131]
PT	ZnO	Electropolymerization growth	Interfacial bonding and morphology control was described	[16]
PEDOT	V_2_O_5_	“Cocoon-to-silk” fiber reeling method	First method for fabricating layered V_2_O_5_/PEDOT nanowires was reported	[132]
	Iron oxide *	Spin-coated-assisted deposition with “supporting layer technique”	A new simple, fast, and inexpensive technique for the fabrication of a free-standing hybrid was reported	[133]
CP **	Ag, Au, CdSe	RAFT polymerization	Role of the direct covalent attachment was emphasized	[134]

* Commercially available chemical; the exact composition is given in the reference; ** a general term, no mention of any specific CPs.

**Table 7:** Representative examples for photosynthesis of CP nanohybrids.

Material	Preparation method	Details	Refs.
PPy/Ag	One-pot UV-induced photopolymerization	Effect of concentration ratio of composite in cellulose fabric was studied	[135]
	One-step interfacial photopolymerization	Thin, flexible nanofilms were synthesized at the water–air interface	[136]
PPy/TiO_2_	Photo-assisted electrodeposition	Effect of LiClO_4_ in the presence of SDBS was investigated	[23]
	Photoelectrochemical polymerization	Properties of nanohybrid in the presence of SDS was studied	[22]
PPy/WO_3_	*In situ* photopolymerization	A room-temperature H_2_S gas sensor was fabricated	[24]
PPy/ceria	Photo-induced polymerization	A good example of one-step photopolymerization was shown	[137]
PPy/methacrylate	UV and visible light photopolymerization	The mechanism of photopolymerization with iodonium salt was presented	[138]
PPy/AgBr/Ag	Microemulsion photopolymerization	Effect of different concentrations of cationic surfactant CTAB was studied	[31]
PT/epoxy	One-pot photoinduced synthesis	A novel methodology for fabricating a network film was given	[20]
Clay/PPy/Ag	*In situ* photopolymerization	The silanization of clay on a PPy/Ag surface was discussed	[139]
PEDOT/TiO_2_	Photoelectrochemical polymerization	Role of donor-π-acceptor sensitizers was described	[140]
		A new method using aqueous micellar solutions was shown	[141]
		The effect of light intensity on the oxidation level of PEDOT was studied	[142]

**Table 8:** Representative examples of studies where carbon nanomaterials were incorporated into CPs.

Carbon	CP	Preparation method	Details	Refs.
GN *	PANI	*In situ* polymerization	A microspherical and porous structure was fabricated	[50]
		Reflux technique	Complex of PANI and GN for enhancing charge-transfer ability was reported	[148]
		Sandwiched GN–mesoporous silica as template	A novel approach to fabricate mesoporous PANI film coating on GN	[149]
		Low-temperature *in situ* polymerization	PANI nanorods were coated on graphene nanomesh	[150]
	PPy	Double-doping electropolymerization	A good example for anchoring double-doped CP on GN sheet was given	[144]
	PEDOT	Electrochemical codeposition	The role of SDS surfactant in the incorporation of GN into PEDOT was studied	[38]
rGO	PANI	*In situ* reduction	A high-surface-area hybrid was reported	[52]
		Electrostatic adsorption synthesis	The thickness was well-controlled by pH modification	[151]
	PPy	Vacuum filtration method	The improvement of cycling stability was demonstrated via the addition of rGO	[152]
		Hydrogen bubble dynamic template	A general method for fabrication of 3D macroporous hybrid was studied	[153]
		Interfacial polymerization	The comparison of two different methods was shown, emphasizing the strength of interfacial polymerization	[154]
		Bioreduction technique	A new simple, environmentally benign method that was time- and cost-efficient was developed	[155]
		One-step synthesis	A good example of a cathode material was shown	[156]
		Interfacial polymerization	A novel electrode material was developed	[157]
	PEDOT	Fast thermal treatment with *in situ* deposition	A good example of a hybrid CP for gas sensing was studied	[158]
SWCNT	PANI	Liquid–liquid interfacial polymerization	The synthesis and characterization of hybrid thin films in liquid–liquid interface was first studied	[159]
	P3HT **	Diels-Alder ligation	A facile covalent strategy was developed to address the bundling issue of CNTs	[160]
MWCNT	PEDOT:PSS	Electrochemical co-deposition	A facile and effective approach for electrode preparation was reported	[54]

* GN—Graphene; ** P3HT—poly(3-hexylthiophene).

**Table 9:** Representative examples of ternary CP nanohybrids.

Ternary system			Details	Refs.
CP	Carbon	Inorganic species		
PANI	GN/CNT		Intercalation of CNT between GN sheets	[163]
			*In situ* deposition of PANI on GN/CNT paper	
	Graphite felt/CNT		Electropolymerization of PANI in the presence of graphite felt	[164]
			Electrophoretic immobilization of CNT on the hybrid	
	GN/CNF *		Production of G/CNF by electrospinning	[165]
			*In situ* polymerization of PANI	
	GO	S	Layer-by-layer synthesis of PANI layer on GO-S composite with heating	[166]
	rGO	MnO_2_	PANI vertically grown on GO sheet	[167]
			Reduction of GO and follow by the deposition of MnO_2_ onto PANI	
	rGO/CNT		Combination of chemical foaming, thermal reduction, and KOH activation to prepare rGO	[168]
			Interfacial polymerization	
	N-doped rGO	NiFe_2_O_4_	NiFe_2_O_4_ simultaneously grown with reduction and doping of GO	[169]
			*In situ* chemical polymerization of PANI	
	CNT	Fe	Reduction of FeCl_3_ in the mixing solution of aniline and CNTs	[170]
	CNT	CoS_1.097_	Hydrothermal synthesis of CNT/CoS_1.097_	[171]
			*In situ* electropolymerization of PANI	
	rGO	MnFe_2_O_4_	Hydrothermal reaction to disperse MnFe_2_O_4_ well on the rGO surface	[172]
			*In situ* polymerization of PANI	
	Vertical rGO	Pd	One-step electrodeposition of PANI and rGO	[173]
			Spontaneous redox reaction of PANI with Pd salt	
	CNT	Ag	*In situ* chemical polymerization of PANI in the presence of AgNO_3_ and MWCNTs	[174]
	C	TiN	Sequential coating of C and PANI on the surface of TiN nanowire array	[175]
	C	S	*In situ* polymerization of aniline on the pore surface of mesoporous C which served as a reservoir for S	[176]
PPy	rGO/CNT		Reduction of GO in the presence of CNT	[177]
			*In situ* chemical polymerization of PPy in the presence of rGO/CNT powders	
	GN	S	*In situ* polymerization of PPy in the presence of GN	[178]
			Suspension mixture of PPy/GN with nano-S	
	CNT	TiO_2_	Chemical preparation of complex of TiO_2_/CNT	[179]
			*In situ* polymerization using MO as template	
PPy/PEDOT	CNT		Series of processes: high-temperature reflux technique, thermal compression, oxygen plasma etching, and electrochemical polymerization	[180]
PT		Pd/TiO_2_	Water-in-oil emulsion of TiO_2_ microspheres	[181]
			Loading Pd species and coating PT	
PEDOT:PSS	rGO	RuO_2_	Mechanical stirring and sonochemical treatment of PEDOT:PSS in PSS-coated rGO solution	[182]
			Chemical interaction between RuO_2_ and hybrid	
	GO/CNT		Self-assembled interfacial coupling method	[123]

* CNF—carbon nanofiber.
